# The effect of rTMS intervention with different targets on neural remodeling in stroke patients: a randomized controlled trial

**DOI:** 10.3389/fneur.2025.1539393

**Published:** 2025-07-17

**Authors:** Li Zhao, Li Chen, Qiu Wang, Xinyi Li, Sha Li, Chunxiao Wan

**Affiliations:** ^1^Department of Rehabilitation Medicine, Tianjin Medical University General Hospital, Tianjin, China; ^2^Department of Medical Imaging, Tianjin Medical University General Hospital, Tianjin, China

**Keywords:** stroke, rTMS, motor cortex, neuronal plasticity, M1, SMA, fMRI

## Abstract

**Background:**

rTMS is widely used to improve motor function in patients with ischemic stroke, but there are few studies on different targets. In order to develop a clinical precision rehabilitation program, this study aims to explore the effects of rTMS at different targets on neural remodeling in patients with subcortical stroke by combining motor function assessment, multimodal MRI and electrophysiological methods.

**Methods:**

69 stroke patients were randomly assigned to the sham group, M1 group, and SMA group. Functional assessment was performed using the exercise and balance scale, and rsfMRI, DTI, VBM, and MEP were used to evaluate the changes in rsFC, white matter tracts, gray matter volume, and neurophysiology before and after intervention.

**Results:**

Following the intervention, the SMA group demonstrated significantly greater improvement in motor function compared to the M1 group (*p* < 0.05). Functional connectivity analysis revealed significantly increased resting-state functional connectivity (rsFC) in the contralateral dentate nucleus and ventromedial premotor area of the affected side in the SMA group relative to the M1 group (*p* = 0.0319), with this enhancement showing a strong positive correlation with balance function improvement (*r* = 0.637, *p* = 0.001). Structural MRI analysis indicated that while the M1 group exhibited a significant increase in gray matter volume (GMV) in the medial segment of the postcentral gyrus (*p* = 0.02), the SMA group showed significant GMV increases in the posterior cerebellum and chorionic lobule (*p* = 0.0428) that demonstrated a moderate positive correlation with improved balance function (*r* = 0.436, *p* = 0.038). Diffusion tensor imaging results showed significant differences in both fractional anisotropy (FA) and apparent diffusion coefficient (ADC) values of the corticospinal tract between the M1 group and the other two groups, with both the M1 and SMA groups exhibiting significant changes in latency and amplitude measures compared to the sham group post-intervention.

**Conclusion:**

High-frequency SMA-TMS intervention on the affected side has a better improvement than traditional M1 target in stroke with motor function. We provide neuroimaging and neurophysiological evidence for different target rTMS interventions in motor related networks after stroke.

**Clinical trial registration:**

www.chictr.org.cn, identifier ChiCTR2200060955.

## Introduction

Stroke is a major health hazard and the leading cause of death and disability in adults, with a trend toward younger age groups (1, 2). Approximately 80% of stroke patients have residual motor dysfunction, which is the main cause of disability and places a heavy burden on the country, society, families, and individuals (3–5).

Neuroplasticity may aid in neural repair (6). Repetitive transcranial magnetic stimulation (rTMS) is a noninvasive magnetic stimulation technique based on the theory of electromagnetic induction. The magnetic field generated by a coil placed at the target penetrates the skull, producing an induced current that stimulates the electrophysiological activity of adjacent nerve tissue. rTMS is widely used in the field of neurological diseases (7), providing regulation through the generation of long-term potentiation (LTP) or long-term depression (LTD) effects. The activity and synaptic transmission of gamma aminobutyric acid (GABA) receptors can exert not only relative focal regulation on the target cortex but also a wider range of diffuse regulation on other brain regions that have functional connections with the target cortex (8, 9). Numerous studies (10–13) have confirmed that high-frequency or low-frequency rTMS combined with other physical rehabilitation treatments can significantly improve stroke motor function recovery. The combination of functional magnetic resonance imaging (fMRI) and other methods has shown that the regulatory mechanisms of rTMS include regulating neural activity in local brain regions (14), altering brain motor network functional connectivity (15, 16), improving neural disconnection (15), regulating the basal ganglia thalamic circuit of the cortex (17), and regulating the effect connectivity of the cortical motor interval (18). These studies have deepened our understanding of the brain remodeling mechanism of rTMS. However, current research is limited by small sample sizes and the different locations, sizes, severities, and courses of lesions. There is a lack of systematic research on stimulus parameters and target settings, and the analysis of brain function between hemispheres and within hemispheres has produced inconsistent results (19–21). The focus of this study was mostly on the primary motor cortex, and there is little comparative research on the auxiliary motor area, especially between the primary motor cortex and the auxiliary motor cortex.

This study explored the mechanism of rTMS in neural remodeling from the perspectives of functional-structural remodeling and neuroelectrophysiology, focusing on multiple indicators, such as resting-state functional connectivity within the motor executive network after stroke, gray matter volume in motor-related areas, corticospinal tract and corpus callosum fiber microstructure, and neurophysiology. This study aimed to clarify the correlation between functional structural electrophysiology and therapeutic effects and to attempt to apply stimulation of different targets to provide a theoretical basis for precise clinical rehabilitation.

## Methods

### Subjects

**Figure 1:**
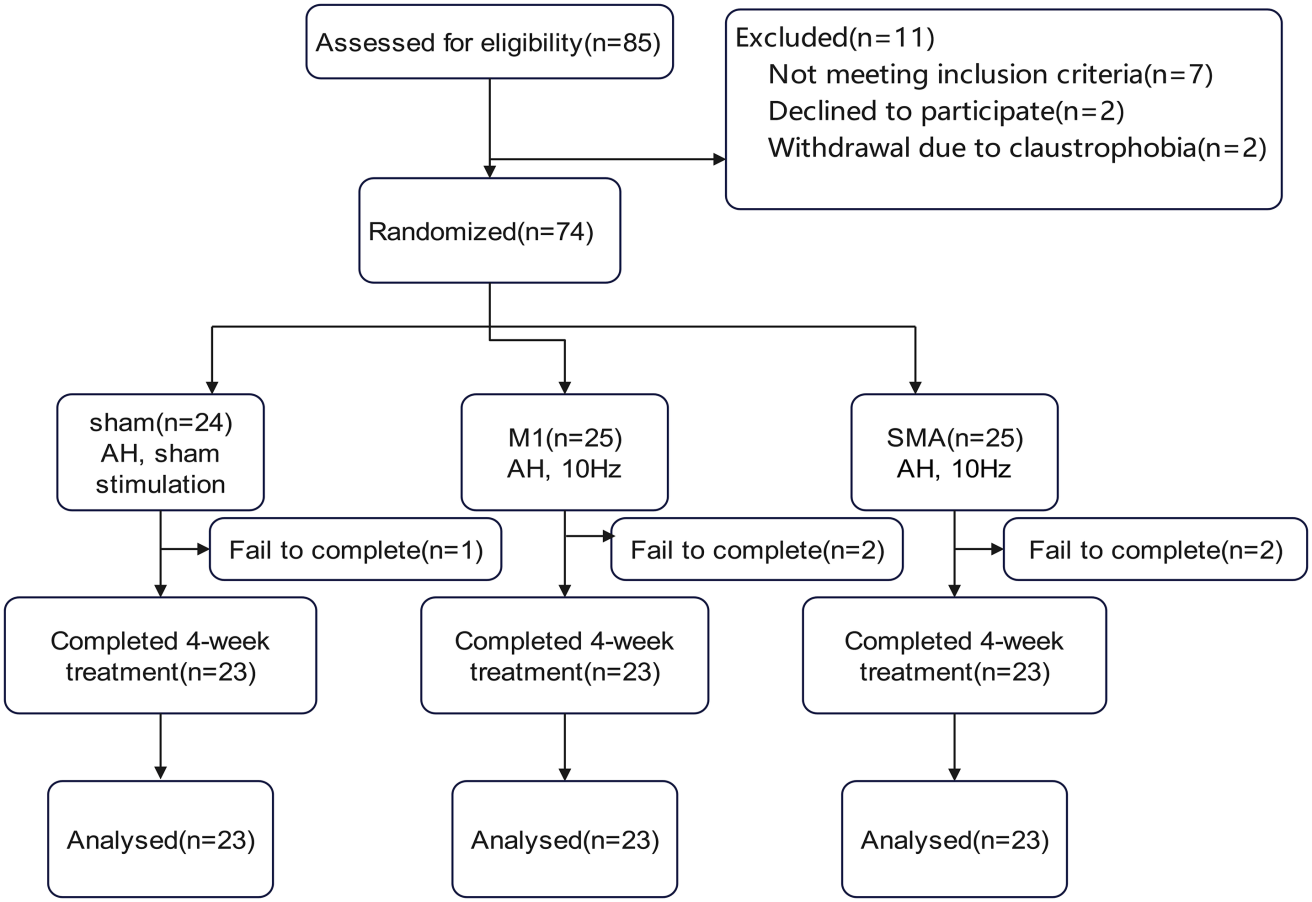
Flowchart of the study procedures.

74 patients with cerebral infarction admitted to the Department of Rehabilitation Medicine of Tianjin Medical University General Hospital from July 2022, to September 2023, were selected as the study subjects (Figure 1). The inclusion criteria were as follows: (1) Patients with subcortical ischemic stroke who met the diagnostic criteria specified in the “Key Diagnostic Points for Various Major Cerebrovascular Diseases in China 2019” formulated by the Neurology Branch of the Chinese Medical Association and have been confirmed by head spiral CT or head magnetic resonance imaging; (2) Initial onset of the responsible lesion, with clinical evaluation showing some degree of motor dysfunction; (3) Within 12 weeks after stroke; (4) Right-handed; (5) No gender restrictions, age from 40–80 years; (6) Stable vital signs, clear consciousness, MMSE>21 points. The exclusion criteria were as follows: (1) Unstable condition, progressive stroke or secondary stroke; (2) Previous peripheral nerve injury or peripheral neuropathy on the affected side; (3) Previous individua history or family history of epilepsy; (4) Severe anxiety or depression symptoms requiring control with medication; (5) Severe heart, lung, liver, kidney or other organ diseases; (6) Presence of a pacemaker, cochlear implant, or skull repair plate in the body; (7) Claustrophobia. The discontinuation and dropout criteria were as follows: (1) Progressive worsening of condition or other newly diagnosed diseases rendering the patient unable to continue treatment; (2) Severe adverse reactions or inability to tolerate rTMS during treatment; (3) Requests by subjects to withdraw for their own reasons.

### Randomization and blinding

Using a simple randomization method, eligible subjects were randomly divided into sham group, M1 group, and SMA group based on their order of entry into the trial, corresponding to the characters in the random number table. All participants provided written informed consent according to the Declaration of Helsinki before the experiment, as approved by the ethics committee of the hospital (approval number: IRB2022-YX-054-01), and registered in the Chinese Clinical Trial Registry (registration number: ChiCTR2200060955).

### Rehabilitation procedures

All subjects received rTMS treatment or pseudo stimulus treatment. The treatment instrument adopts Mag TD 100 Hz magnetic field stimulator (Wuhan Yiruide Medical Equipment Co., Ltd., Wuhan, China). During treatment, the subject is placed in a comfortable sitting or supine position, with an “8-shaped” coil placed at the subject’s stimulation target. The coil is tangent to the surface of the skull during treatment, and the parameters were as follows: rTMS stimulation with a stimulation frequency of 10 Hz and an intensity of 100% RMT, with a stimulation frequency of 2.5 s, an interval of 10 s, once a day, for 20 min each time, a total of 2,400 pulses, 5 days a week, last for 4 weeks. The control group received rTMS pseudo simulation. During the study period, all subjects received routine drugs, physical therapy, occupational therapy and acupuncture and moxibustion. Using the 10–10 international EEG standard to locate stimulation targets (M1: midpoint of the line connecting CPz to C3 or C4; SMA: FC1 or FC2; Sham: Midpoint of Pz and C3/C4).

### Output

The entire research process lasted for four weeks, and the following evaluations were given before and after intervention.

Main outcome: Motor function assessment. The upper and lower limb Fugl Meyer motor function scale (FMA) and balance function scale (BBS) were used to evaluate the patient’s upper and lower limb function and balance function, respectively.

Secondary outcomes include functional, structural, and neurophysiological indicators. Using Resting State Functional Connectivity (rsFC) based on Region of Interest to describe the functional connectivity of the motion execution network; Gray matter volume (GMV); Monitor changes in white matter microstructure by measuring the anisotropy fraction (FA) and apparent diffusion coefficient (ADC) of bilateral corticospinal tract (CST) and corpus callosum (CC); Use TMS-MEP to monitor the resting period and amplitude changes of motor evoked potentials.

### MRI data acquisition

Resting state functional magnetic resonance data acquisition: Functional and structural MRI data were obtained using the 3.0 Tesla Siemens Trio Tim system and 12 channel magnetic head coils. All subjects’ heads were fixed with customized foam pads to reduce head movement. During the functional magnetic resonance imaging scan, the subjects were instructed to relax, remain stationary, and close their eyes. Parameter settings: (1) Use high-resolution gradient echo echo planar imaging (GRE-EPI) technology to scan functional imaging in resting state and observe the functional connections between different brain regions in the motor execution network during resting state. The parameters are as follows: Repetition Time: 800 ms, Echo Time: 30 ms, Flip Angle: 56 °, FoV 100 × 100 mm^2^, Matrix size 104 × 104, Slice Thickness: 2 mm, Number of slices: 72. (2) Using 3D BRAVO sequence sagittal scanning, high-resolution T1WI anatomical images of the entire brain were obtained. The parameters are as follows: T*R* = 2000 ms, TE = 2.32 ms, FA = 8 °, FOV = 256 mm × 256 mm, matrix = 256 × 256, slice thickness = 0.9 mm, and slices = 208; And (3) DTI images were observed by a diffusion weighted pulsed gradients spin EPI sequence: T*R* = 3,900 ms, TE = 90 ms, FA = 90°, FOV = 105 mm × 105 mm, matrix = 140 × 140, slice thickness = 1.5 mm, voxel size = 1 mm × 1 mm × 1 mm, slices = 193.

### Image data preprocessing

Preprocess the data using MATLAB (R2014b, The Math Works, Natick, MA, USA) software and DPARSFA. For each participant, the first 20 time points of data were excluded to avoid scanner instability and enable participants to adapt to scanner noise. The remaining 430 time points of data were preprocessed. Before data preprocessing, first flip the image of the lesion located in the left hemisphere to the right, with the right hemisphere uniformly defined as the affected hemisphere and the left hemisphere as the healthy hemisphere. Preprocessing includes: time correction, head movement correction, spatial standardization, spatial smoothing, de linear drift, low-frequency filtering (0.01 ~ 0.1 Hz), and removal of physiological interference factors (head movement, whole brain signal, white matter signal, and cerebrospinal fluid). Based on ROI functional connectivity, calculate the rsFC between the ROIs in the motion execution network.

Definition of seed points: According to Wang et al. (22) definition of the motor execution network, the lesion locations (basal ganglia and thalamus) of the included subjects were excluded, and 9 seed points on each side were obtained to form the motor execution network (Supplementary material 1).

Perform voxel based morphological measurement (VBM) on 3DT1 using SPM12. The brain image undergoes an initial segmentation process, and the DARTEL toolbox in SPM12 is used to continuously estimate the average template of its group gray matter (GM). Then use the final DARTEL transformation to standardize the GM segmentation and modulate it according to volume changes. Finally, use a 10 mm full width half width Gaussian kernel to smooth the image. Statistical analysis was conducted using SPM12 using whole brain volume (TIV) as a covariate.

Use FSL software to perform head movement and eddy current correction on the image data converted from DTI data, remove the skull and scalp, perform tensor reconstruction, and perform linear registration. Manually draw the fibers of interest using TrackVis and display the average FA and MD values. For all fiber bundle reconstruction, the algorithm settings are as follows: the threshold for FA is set to 0.2, and the angle threshold is set to 45°.

### Statistics

This study is a randomized controlled trial, divided into M1 group, SMA group, and Sham group, with a = 0.05, 1- *β* = 80%, effect size = 0.4. The one-way ANOVA F-test under the mean menu of PASS 2021 software was used, and the total sample size was 66 cases. Considering the number of cases of loss to follow-up and refusal to follow up, calculated at 20%, 66 x (1 + 0.2) = 79.2 cases. A total of 85 cases were included in this study, with 16 cases dropped out. Finally, 23 cases were included in each group for statistical analysis.

SPSS 27.0 statistical analysis software was used for data processing, and(𝑥̅±𝑠)was used for quantitative data analysis represents the baseline age of the subjects, functional assessment, gray matter volume extracted from areas of interest related to motor activity, FA and ADC values of the corticospinal tract and corpus callosum, and MEP values compared within the group before and after treatment using paired sample t-tests for analysis; Multiple group comparisons were conducted using one-way analysis of variance (ANOVA); The subsequent comparison between two groups was analyzed using the Least Significant Difference (LSD) method, and the difference was statistically significant at the test level of *p* < 0.05. SPM12 software was used to perform paired t-tests, one-way ANOVA, and *post hoc t*-tests on rsFC and GMV values using a general linear model to obtain significant differences (*p* < 0.05, Bonferroni correction). Pearson correlation analysis and partial correlation statistical analysis are used to measure the relationship between primary outcomes and imaging biomarkers. Visualize the results using Xjview and Brainnet view software.

## Result

### General situation of subjects and evaluation baseline

A total of 69 people completed the entire research process. There was no statistically significant difference in demographic characteristics and clinical evaluation baseline among the three groups of subjects (Table 1).

**Table 1:** General situation and baseline evaluation of included subjects.

Category	Sham (*n* = 23)	M1 (*n* = 23)	SMA (*n* = 23)	*F*	*P*
Age	63.221 (8.919)	61.029 (7.013)	59.778 (10.072)	0.911	0.407
Female (*n*)	13	14	14		
Right handedness (*n*)	23	22	23		
Duration (day)	35.003 (17.211)	30.089 (19.333)	33.711 (17.015)	0.467	0.629
Left/right hemisphere (*n*)	10/13	11/12	9/14		
FMA-UE	14.913 (6.508)	15.043 (4.385)	16.478 (4.757)	0.618	0.542
FMA-LE	14.174 (5.433)	15.000 (6.038)	15.870 (4.902)	0.551	0.579
BBS	12.696 (4.884)	12.348 (6.658)	12.378 (6.582)	0.025	0.975

FMA-UE, Fugl-Meyer motor assessment scale-Upper Limb; FMA-LE, Fugl-Meyer motor assessment scale-Lower Limb; BBS, Berg Balance Scale. Data are presented as mean (standard deviation).

**Figure 2:**
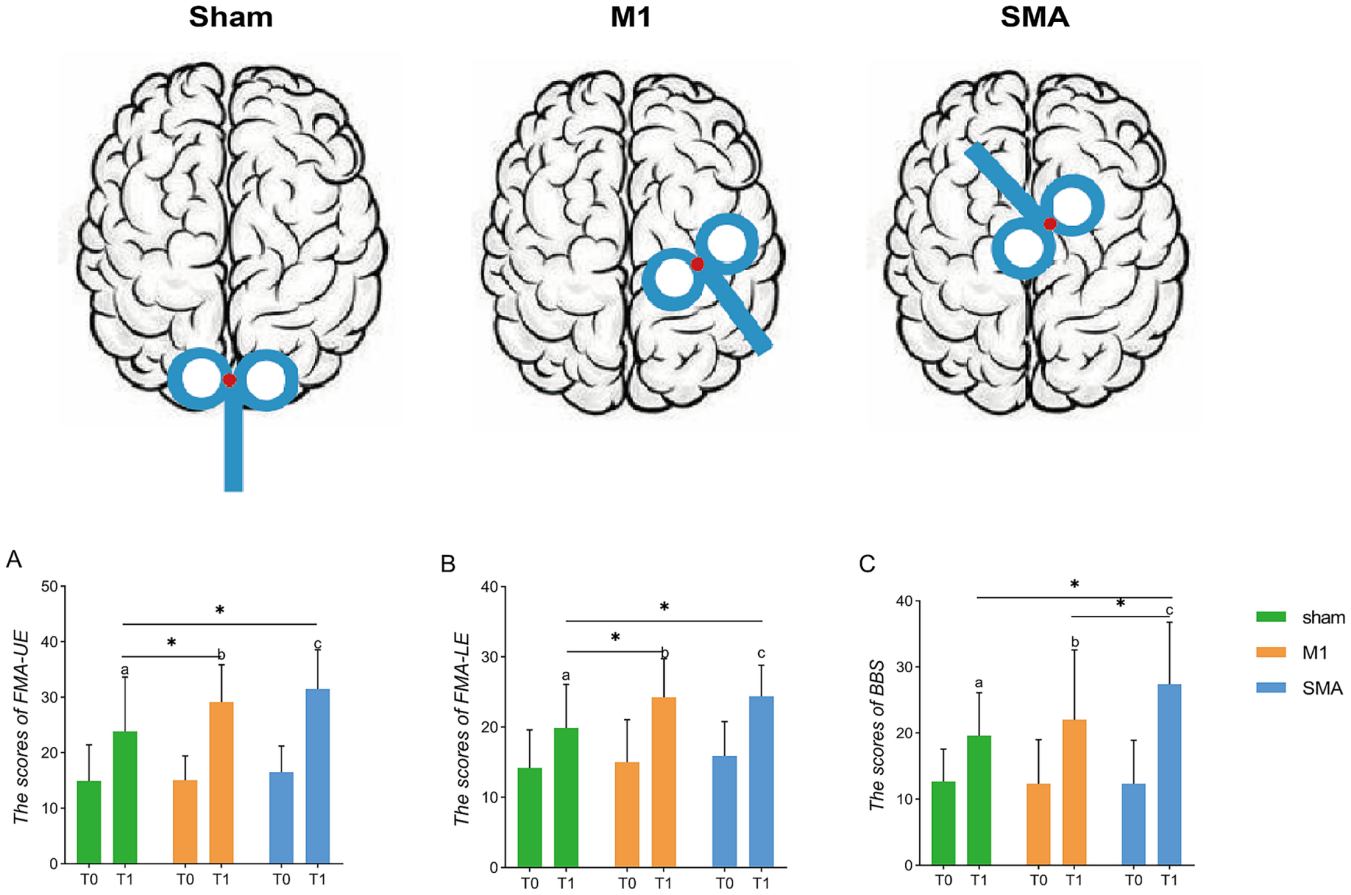
Assessment of function. Schematic diagram of rTMS stimulation targets. **(A–C)** Assessment of upper and lower limbs and balance function. ^*^p < 0.05; **(A–C)**: *p* < 0.001, respectively, show the three groups before and after treatment. T0, baseline; T1, after treatment.

After treatment (T1), the scores of the upper limbs (FMA-UE, Figure 2A), lower limbs (FMA-LE, Figure 2B), and balance function (BBS, Figure 2C) of the three groups were all improved compared to baseline (T0) (p_abc_<0.001). There was a significant difference in the analysis of variance among the three groups after treatment (*F* = 5.470, *p* = 0.06; *F* = 5.171, *p* = 0.008; *F* = 4.522, *p* = 0.014). *Post hoc* LSD test showed that compared with the Sham group, the 4-week SMA-rTMS group had significant increases in FMA-UE, FMA-LE and BBS scores (*p* = 0.002; *p* = 0.006; *p* = 0.005). The motor function of upper and lower limbs (FMA-UE, FMA-LE) was significantly improved in the M1-rTMS group (*p* = 0.021; *p* = 0.008). The BBS score of SMA group was significantly higher than that of M1 group (*p* = 0.046).

### rsFC within the motor execution network

**Figure 3:**
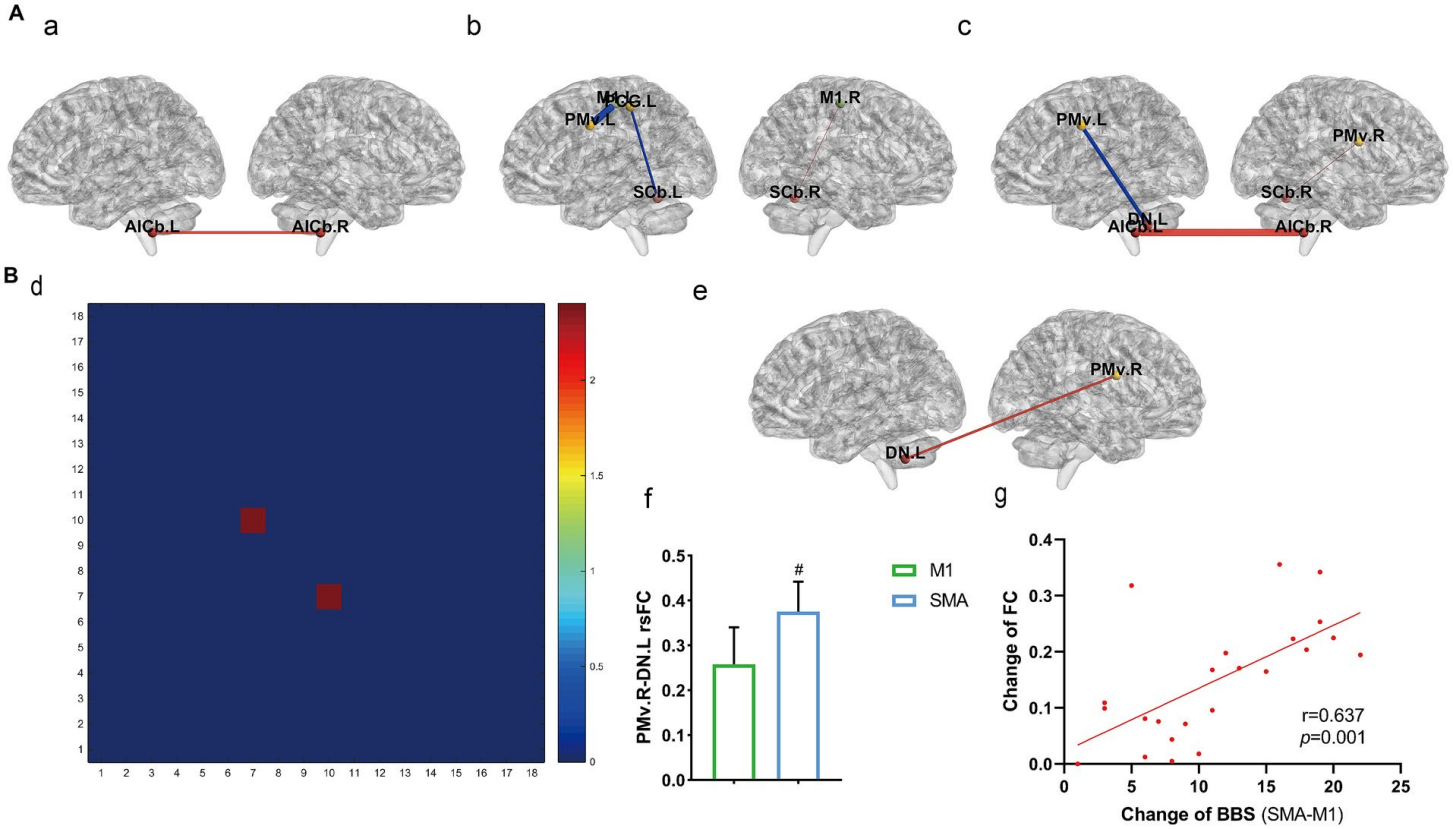
rsFC within the motor exeution network. **(A)** rsFC changes before and after treatment among the three groups **(a)** sham group, **(b)** M1 group, **(c)** SMA group. **(B)** rsFC comparison among the three groups after treatment; **(d)** Three-group analysis of variance (1–18: 18 brain regions, see Supplementary material 1 for details); **(e,f)** results of *post-hoc t*-test for SMA and M1 groups; **(g)** correlation analysis between fc changes and BBS. #*p* < 0.05.

Comparison of rsFC among the three groups before and after treatment was also performed (Figure 3A). The sham group showed enhanced rsFC in the anterior inferior cerebellum on both sides (a). In the M1 group, the primary motor area on the affected side (right) and the ipsilateral superior cerebellum showed increased rsFC, while the primary motor area and ventrolateral premotor cortex on the healthy side (left), as well as the postcentral gyrus and superior cerebellum, showed decreased rsFC (b). The SMA group showed increased rsFC in the ventrolateral premotor cortex and superior cerebellum on the affected side, as well as in the lower part of the bilateral cerebellar anterior lobe. The rsFC in the ventrolateral premotor cortex and dentate nucleus on the healthy side decreased (c) (*p* < 0.05, Bonferroni correction).

Comparison of rsFC among the three groups after treatment was also performed (Figure 3B). There was a significant difference in rsFC between the left primary motor area and the right dentate nucleus according to three sets of analysis of variance (d). A *post hoc t* test revealed that the SMA group had enhanced rsFC in the left dentate nucleus and right ventromedial anterior motor area compared to the M1 group (*p* = 0.0319, e, f), and the difference in BBS between the two groups (y-axis, g) was strongly positively correlated with the change in M1. L-DN. R rsFC (x-axis, g) (*r* = 0.637, *p* = 0.001; Pearson correlation analysis).

### Volume of gray matter (GMV)

**Figure 4:**
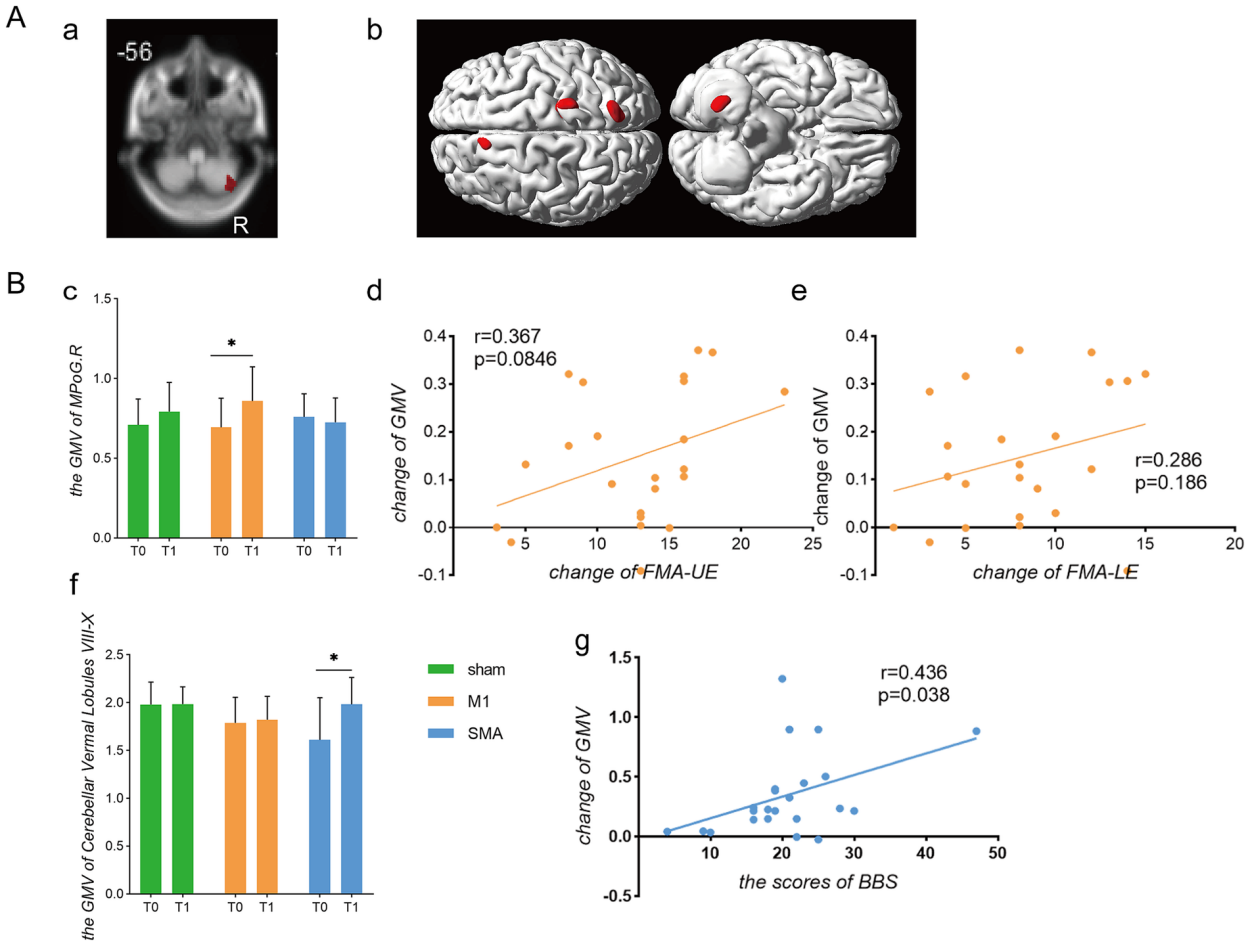
Comparison of gray matter volume changes. Gray Matter Volume **(A)** Brain regions with increased gray matter volume. **(a)** Brain regions showing changes in gray matter volume before and after intervention in the SMA group; **(b)** Brain regions showing differences in gray matter volume among the three groups after intervention. **(B)** Analysis of gray matter changes and their correlation with functional scores. **(c)** Gray matter volume in the contralateral postcentral gyrus; **(d,e)** Correlation analysis between changes in gray matter volume in the contralateral central gyrus and FMA-UE/FMA-LE scores, respectively; **(f)** Gray matter volume in cerebellar vermis lobules VIII-X; **(g)** Correlation between changes in cerebellar gray matter volume and changes in BBS scores.*p < 0.05.

Comparison of voxel-based morphometry (Figure 4A) showed only an increase in GMV of the right cerebellum after intervention in the SMA group (*p* = 0.01, uncorrected, Figure 4A). After intervention, the analysis of variance of all three groups showed changes in the left middle frontal gyrus, right posterior central gyrus, right parietal lobe, and right cerebellum (*p* = 0.01, uncorrected, Figure 4B). Comparing the GMV values extracted from motor related brain regions (Figure 4B), it was found that the M1 group had an increase in the right central posterior gyrus after intervention compared to before intervention (*p* = 0.02, Figure 4c). The changes were correlated with the upper and lower limb motor function scores of the group, but there was no significant correlation (*r* = 0.367, *p* = 0.0846; *r* = 0.286, *p* = 0.186; Figures 4d,e). Before and after intervention in the SMA group, it was found that there was an increase in the vermiform lobule VIII-X of the cerebellum, namely the posterior lobe of the cerebellum and flocculonodular lobe (*p* = 0.0428). Correlation analysis showed that this change was a moderate positively correlated with the improvement of balance function in the group (*r* = 0.436, *p* = 0.038; Figures 4f,g).

### White matter fiber microstructure and neurophysiology

**Figure 5:**
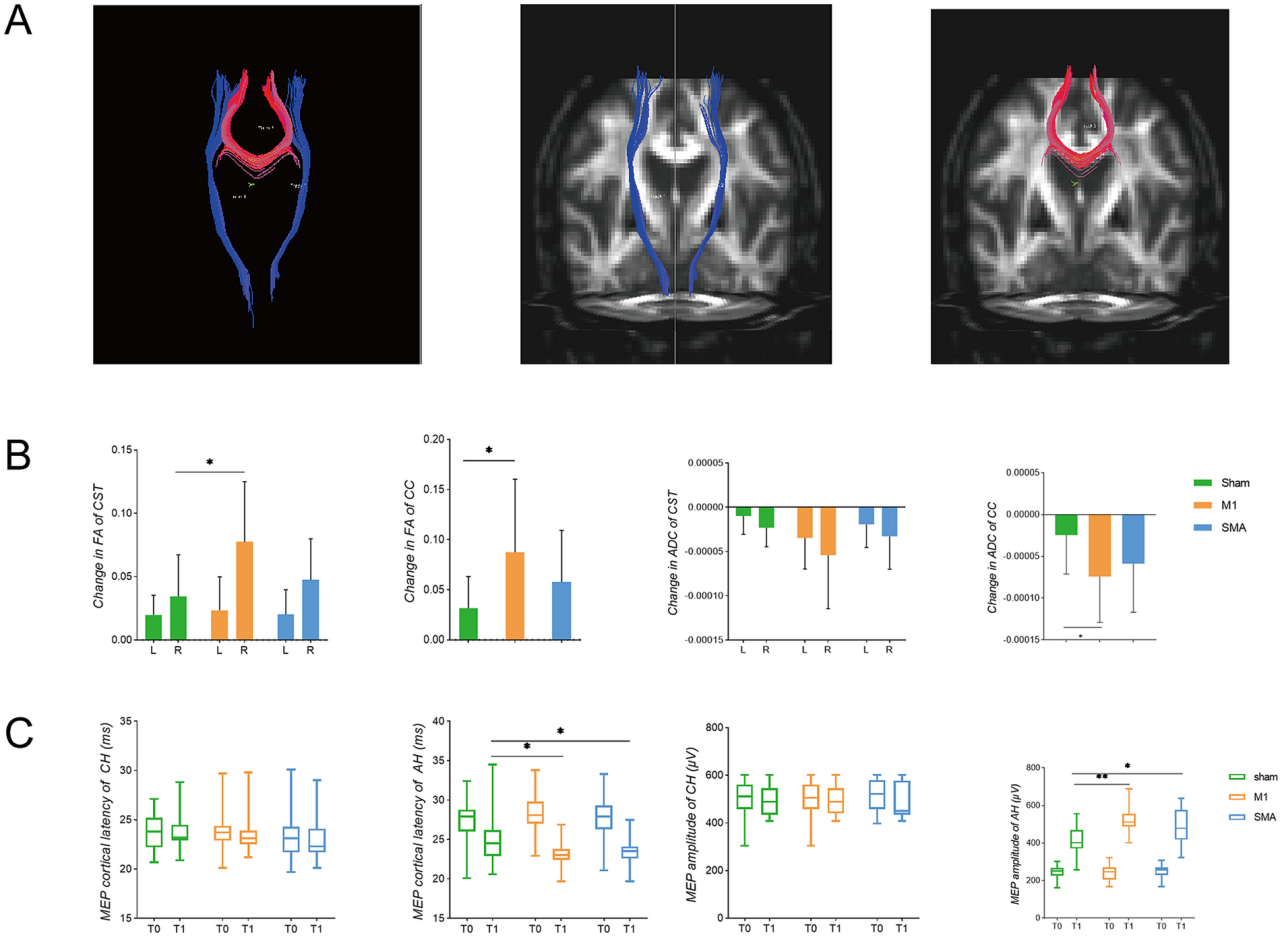
White matter fibers and electrophysiological changes. **(A)** fiber bundle for deterministic tracking. Red represents the corpus callosum (CC), and blue represents the corticospinal tract (CST); **(B)** Changes in FA and ADC of white matter fibers. L left, R right (affected side); **(C)** TMS-MEP latency and amplitude changes. T0: before intervention, T1, after intervention; CH, contralateral hemisphere; AH, affected hemisphere. *p < 0.05; **p<0.01.

Fiber bundle tracking (Figure 5A). Compared to before intervention, the FA and ADC of the affected corticospinal tract and corpus callosum in all three groups of subjects improved. The FA values of the corticospinal tract and corpus callosum in the M1 group were significantly improved compared to the sham group (*p* = 0.0276, *p* = 0.0383), while the ADC values of the corpus callosum were significantly reduced compared to the sham group (*p* = 0.0386) (Figure 5B). In terms of electrophysiology, both the M1 group and the SMA group showed improvements in the latency and amplitude of motor evoked potentials compared to the sham group. In the M1 group, the latency was significantly reduced (*p* = 0.0084) and the amplitude was significantly increased (*p* < 0.0001); SMA group: The latency significantly decreased (*p* = 0.0393) and the amplitude significantly increased (*p* = 0.0036) (Figure 5C). However, no significant difference was found between the M1 group and the SMA group after intervention.

### Correlation analysis

**Figure 6:**
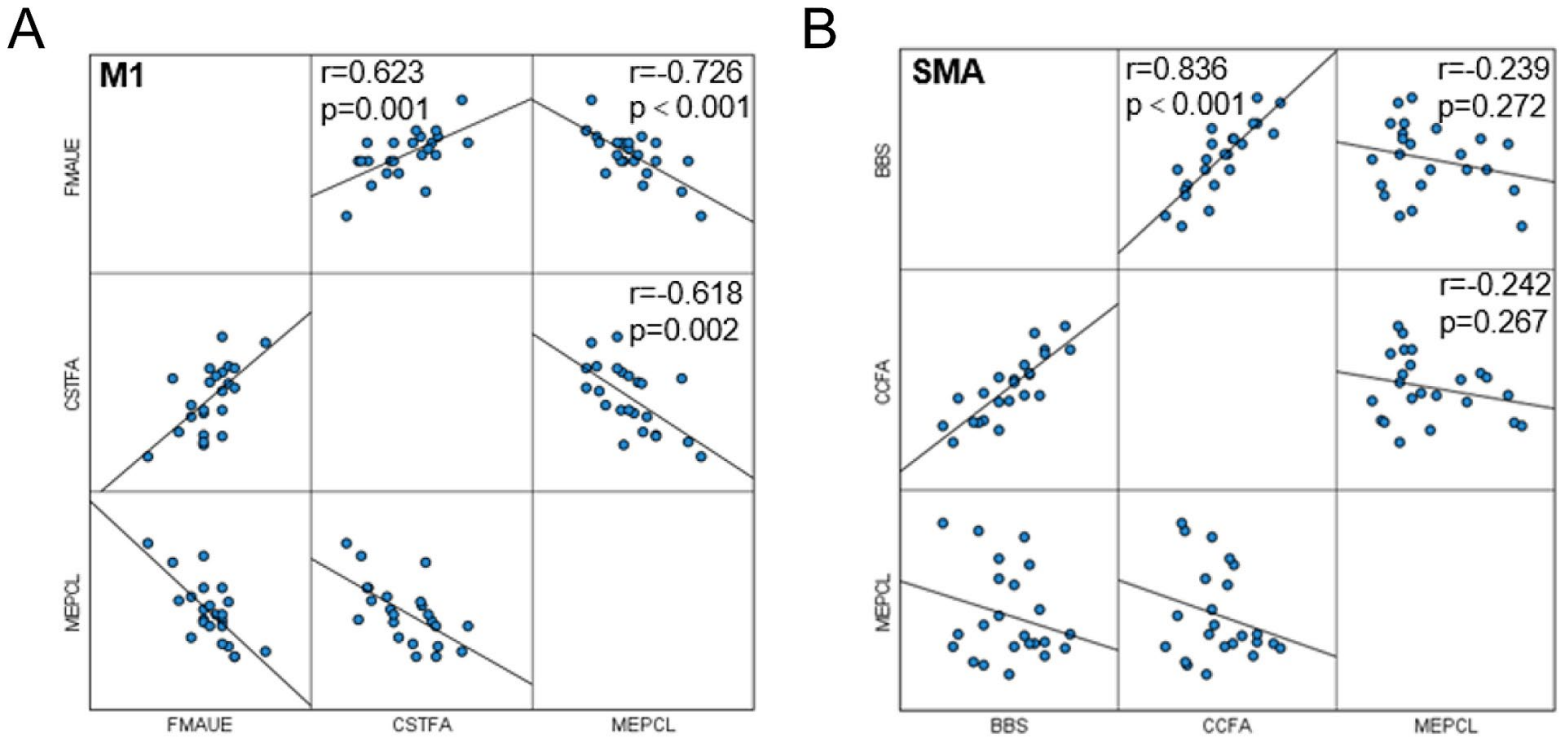
Correlation analysis. **(A)** Correlation between MEP latency, FA of corticospinal tract and FMA-UE in M1 group; **(B)** Correlation between MEP latency, FA of the corpus callosum and BBS in the SMA group.

Functional evaluation and partial correlation analysis between white matter fiber bundle integrity and conduction velocity showed that there was a correlation between the upper limb motor function score before and after intervention in the M1 group and the FA value and MEP latency of the affected corticospinal tract (Figure 6A); The SMA group showed a positive correlation between changes in balance function score and changes in FA value of the corpus callosum (Figure 6B). No significant correlation was found in the remaining correlations.

## Discussion

Eighty percent of stroke patients still have motor dysfunction, and the incidence of motor dysfunction is trending toward younger age groups, which places an enormous burden on families and society (1–4). The rate of brain nerve repair is the fastest from a few hours to approximately 10 weeks after stroke, after which it gradually slows down and reaches a plateau 3 months after stroke (23). Therefore, developing and implementing precise and personalized rehabilitation plans within an effective time frame can not only increase rehabilitation efficiency but also reduce economic burden. This was the original intention of our study, as we hoped to provide a theoretical basis for the development of clinical precision rehabilitation plans.

The pathogenesis of ischemic stroke is complex and involves mainly excessive excitatory amino acids, oxidative stress, calcium overload, inflammatory response, and cell apoptosis (24–26). Subsequently, loss of white matter fiber bundles, loss of neural function, and imbalance of bilateral hemispheric inhibition occur. In this study, we applied multimodal imaging and electrophysiological techniques to explore the role and clinical efficacy of repetitive transcranial magnetic stimulation of different targets in the recovery of motor function after stroke from the perspectives of neural remodeling structure, function, and electrophysiology. The present study revealed that (1) interventions at different targets have different effects on motor function recovery, and SMA target stimulation on the affected side has a better effect on improving balance function; (2) after a stroke, there is compensation in the nondamaged hemisphere, and as the patient recovers, this compensatory effect weakens, and instead, the damaged hemisphere regains function; and (3) neural remodeling involves multiple aspects, such as functional connectivity, gray matter volume, and changes in the microstructure of white matter fibers, as well as electrophysiological changes.

### Clinical efficacy

According to the 2020 updated European TMS evidence-based guidelines, low-frequency and high-frequency rTMS for treating motor dysfunction after subacute stroke are recommended at the A and B levels, respectively, while low-frequency rTMS for treating motor dysfunction after chronic stroke is recommended at the C level (27). This study is based on the “interhemispheric competition” model (28), which suggests that the two hemispheres are interconnected and mutually inhibited through the corpus callosum. The corpus callosum not only transmits information between the two hemispheres but also regulates the balance between the hemispheres. The synergistic effect of excitation and inhibition of the corpus callosum results in a dynamic balance of excitability in both hemispheres. After a stroke, the balance between the two hemispheres is disrupted, and the cortical excitability of the affected hemisphere is not only reduced due to self-injury but also inhibited by the opposite hemisphere; that is, the excitability of the affected hemisphere is “double inhibited.” This model is based on the imbalance between hemispheres after stroke and assumes that correcting the imbalance of excitability between hemispheres after injury is beneficial for brain function remodeling, that is, reducing the excitability of the healthy motor cortex through low-frequency rTMS or upregulating the excitability of the affected motor cortex through high-frequency rTMS. This study is based on that theory and describes high-frequency rTMS intervention in different motor cortices in the affected hemisphere. Previous studies (29–31) have shown that TMS plays a significant role in improving motor function recovery, as indicated by changes in the upper and lower limbs, balance, and gait after stroke. This study is the first randomized controlled study on different targets (M1 and SMA). The results showed that the two groups treated with rTMS intervention after stroke showed significant improvement in limb movement and balance function recovery compared to the control group. Importantly, compared to those in the M1 group, the participants in the SMA group had a significant improvement in balance function.

Based on these results, we conducted a systematic study on the brain network function, structure, and electrophysiology of the three groups of participants, attempting to discover the regulatory effects of different targets of rTMS in the process of neural remodeling.

### The effect of rTMS on brain functional connectivity

Resting-state functional magnetic resonance imaging (rs-fMRI) is a noninvasive brain imaging method that can detect the anatomical and functional connections between various brain regions connected by synapses, as well as the plasticity between networks. In this study, we monitored the functional connections between brain regions within the motor execution network from the perspective of a functional brain network composed of interconnected brain regions. The motor execution network involves the primary motor cortex, premotor area, auxiliary motor area, central posterior gyrus, superior parietal lobule, and some structures of the cerebellum; participates in the perception, planning, preparation, execution, and coordination of movements; and plays an important role in the process of motor recovery. Previous studies (32) have shown that activation of the healthy motor area is enhanced after stroke, and the functional connectivity between the affected and healthy sides is significantly increased. It is speculated that this strategy may be used to compensate for the functional decline caused by the injury. On the other hand, the enhanced functional connectivity of the healthy side may also be due to the weakened inhibition of the affected side on the healthy side. This study revealed that both groups of patients receiving rTMS intervention exhibited a decrease in rsFC in the motor execution network brain regions and an increase in rsFC in the affected areas, indicating a decrease in compensation on the healthy side after stroke and a gradual onset of functional improvement in the affected brain region. This study also revealed an increase in rsFC between the bilateral cerebellum and between the cerebellum and other motor brain regions, reflecting the role of the cerebellum in motor function integration in the motor executive network. Specifically, the M1 group showed an increase in rsFC between the affected primary motor cortex and cerebellum, while the SMA group showed an increase in rsFC between the affected premotor area and cerebellum. Taken together, these findings indicate that rTMS intervention can reduce functional compensation in the healthy side in the early stage of stroke and enhance functional recovery in the affected side; this study also verifies the results of previous studies that large-scale neural network recombination between the cortex and subcortical regions, as well as the cerebellum, after chronic stroke contribute to motor recovery and motor relearning after stroke (22).

The dentate nucleus is the largest nucleus in the cerebellum and is involved in the planning and execution of voluntary movements, as well as in the transmission of sensation. The dentate nucleus receives ascending projection fibers from the spinocerebellar tract and transmits proprioception about the length and tension of the muscle fiber tracts through the lower cerebellar peduncle. It also receives descending fibers from the pre-exercise area and the auxiliary exercise area, which are related to the planning and initiation of casual exercise. By integrating these two types of incoming fibers, the dentate nucleus participates in maintaining balance and coordination in the body. Its efferent fibers pass through the upper cerebellar peduncle and red nucleus, reach the ventrolateral nucleus of the thalamus, and participate in the timing and fine-tuning of voluntary movements. After treatment in this study, analysis of variance and *post hoc* t tests were performed for three groups of subjects. Compared with the M1 group, the rsFC of the healthy dentate nucleus and the affected ventrolateral premotor area were greater in the SMA group, and the difference in rsFC and balance function score between the two groups was positively correlated, indicating its role in balance recovery. The healthy dentate nucleus and the affected ventrolateral premotor area may pass through up- and downfiber conduction, respectively, regulating physical balance and coordination. The M1 group showed changes in FC mainly in the lateral hemisphere, while the SMA group showed changes in some interhemispheric regions, especially in the cerebellum between the two hemispheres (Figure 3). Presumably, M1 and SMA stimulation drive different plasticity mechanisms -unilateral motor recovery vs. Bilateral network rebalancing - Advancing personalized neuromodulation for stroke rehabilitation.

### The effect of rTMS on gray matter volume in the brain

A study comparing the changes in gray matter volume after stroke in patients grouped according to stroke location showed that GMV was reduced in both the ipsilateral sensorimotor cortex of patients with inner capsule stroke and the cerebellum of patients with pontine stroke (33). It is speculated that these changes are due to the dense anatomical connection between the lesion and its corresponding atrophic area (i.e., the corticospinal tract connecting the inner capsule lesion and the sensory motor cortex or the connection between the pons lesion and the cerebellum) (34). In this study, a general linear model was used to compare the gray matter volume of the cerebellar cortex, parietal lobe, and posterior central gyrus before and after treatment in three groups of subjects. However, these changes were not corrected. Subsequently, we extracted gray matter volume values from motor-related areas, such as the bilateral anterior and posterior gyrus, medial segment of the anterior and posterior gyrus, auxiliary motor area, superior parietal lobule, fusiform gyrus, and cerebellum, for comparison. We found significant differences in gray matter volume in the medial segment of the right posterior central gyrus in the M1 group before and after treatment. The central posterior gyrus is the area where the primary sensory cortex is located, and the medial segment is involved mainly in the sensory function of the lower limbs. However, we did not find any correlation between these volume changes and upper or lower limb motor activity. The SMA group showed a significant increase in gray matter volume in cerebellar lobules VIII to X after treatment compared to before treatment. The cerebellar lobules VIII to X are composed of a portion of the posterior cerebellar lobe and the chorionic lobules. The posterior cerebellar lobe affects the initiation, planning, and coordination of movement, while the chorionic lobules participate in adjusting muscle tension and maintaining body balance. *Post hoc* correlation analysis revealed that the change in gray matter volume in this area was positively correlated with the balance function score of the SMA group, which also confirmed the important role of this area in balance function. It is speculated that the different regions with increased gray matter volume may also be related to the differences in anatomical connections between M1 and SMA targets involved in structural remodeling.

### The effect of rTMS on the microstructure of white matter fibers in the brain

DTI (35, 36) monitors the integrity and directionality of white matter fiber bundles in the brain through the diffusion characteristics of water molecules, and various anisotropy indices and average diffusion coefficients are commonly used as evaluation indicators for DTI. FA represents the degree of directional diffusion of water molecules in different tissue microstructures and ranges from 0 (maximum isotropy) to 1 (maximum anisotropy). The higher the FA value is, the greater the degree of directionality and the stronger the neural conductivity. Changes in FA parameters can reflect the formation, destruction, loss, and repair of axonal myelin sheaths and can be used to evaluate the degree of secondary neurodegeneration and white matter fiber bundle damage after stroke. The apparent diffusion coefficient (ADC) reflects the range of diffusion motion of water molecules per unit of time, and the larger its value is, the stronger the diffusion ability of water molecules. The corticospinal tract and corpus callosum are fiber bundles involved in motor function that descend from the hemisphere and connect the left and right hemispheres. They play a significant role in information transmission and affect final behavioral outcomes (37). The corticospinal tract originates from different regions, including the primary motor area (M1), primary sensory area (S1), premotor area, and auxiliary motor area (SMA). Corticospinal tract injuries from different sources may lead to different functional impairments. A study (38) showed that the isotropy of M1 fibers in healthy adults is related to walking endurance, while the isotropy of SMA fibers is related to exercise flexibility. M1 is considered the main origin of the CST, and its impact on motor function seems to be more important than that of other CST fibers (39, 40). This study yielded similar results, revealing that the M1 group exhibited a more significant impact on the microstructure of the corticospinal tract and corpus callosum. However, in this study, we did not find any differences in the corticospinal tract between the M1 group and the SMA group. A possible reason is that in this study, we included changes in the entire corticospinal tract and did not refine the statistics of corticospinal tracts from different sources. Further research on corticospinal tracts from different sources may compensate for our shortcomings and better explain the results.

### The effect of rTMS on motor-evoked potentials

Motor-evoked potentials can objectively reflect the excitability of the motor cortex and quantitatively evaluate central motor conduction function and have been proven to serve as biomarkers for predicting the prognosis of motor function after stroke (41). The conduction pathway of the MEP is the corticospinal tract, and the length of the latency reflects the nerve conduction velocity. Damage to the corticospinal tract after stroke not only damages the conduction structure but also causes varying degrees of damage to electrophysiological characteristics, leading to an extension of the latency period. The amplitude reflects the number of firing neurons. The amplitude of MEPs in normal individuals varies greatly, and the repeatability of MEPs recorded at different stages in the same person is only 63–94%. The amplitude of the MEP on the hemiplegic side of stroke patients is generally lower than that on the healthy side. Research has found that stimulating M1 with iTBS can enhance the amplitude of MEPs (42). Similar results were obtained in this study: the latency was shorter and the amplitude was greater after rTMS intervention than before treatment. There were significant changes in both the M1 and SMA groups, but there was no significant difference between the two groups. Correlation analysis of functional parameters, white matter fiber bundle integrity, and conduction velocity revealed significant correlations between the upper limb functional score of the M1 group and the FA value of the affected corticospinal tract and the latency of motor-evoked potentials. The balance function score of the SMA group was correlated with the FA value of the corpus callosum. Moreover, the different effects of rTMS at two different targets on motor function may be exerted through different pathways.

In summary, this study demonstrated that rTMS promotes the improvement of motor function in stroke patients. The clinical efficacy of rTMS intervention at different targets varies, which may be related to the different pathways involved in neural remodeling. M1 may focus on the motor cortex corticospinal tract, while the SMA may focus on the structural and functional connection between the motor cortex and the corpus callosum cerebellum. The coupling of brain network structure and function neurophysiology deserves further research. This study revealed that SMA stimulation is more effective at restoring balance function than other targets and could provide a theoretical basis for further research on precision rehabilitation.

### Limitations

This study intervened for four weeks without a longer follow-up period to observe the sustainability and long-term efficacy of the intervention. On the other hand, our study only focused on motor related brain networks, gray matter volume, and white matter fibers, which may have overlooked the role of other tissue structures in motor function remodeling, losing some important information to better explain the neural remodeling mechanism of rTMS.

## Conclusion

High-frequency SMA-TMS intervention on the affected side has a better improvement than traditional M1 target in stroke with motor function. We provide neuroimaging and neurophysiological evidence for different target rTMS interventions in motor related networks after stroke.

## Data Availability

The original contributions presented in the study are included in the article/Supplementary material, further inquiries can be directed to the corresponding author.
