# Supplementary figures and images for: The effect of rTMS intervention with different targets on neural remodeling in stroke patients: a randomized controlled trial
Source: Front Neurol. 2025 Jul 17;16:1539393. doi: 10.3389/fneur.2025.1539393 (PMC12310651; doi:10.3389/fneur.2025.1539393)

supplementary material 1

**Motion Execution Network**


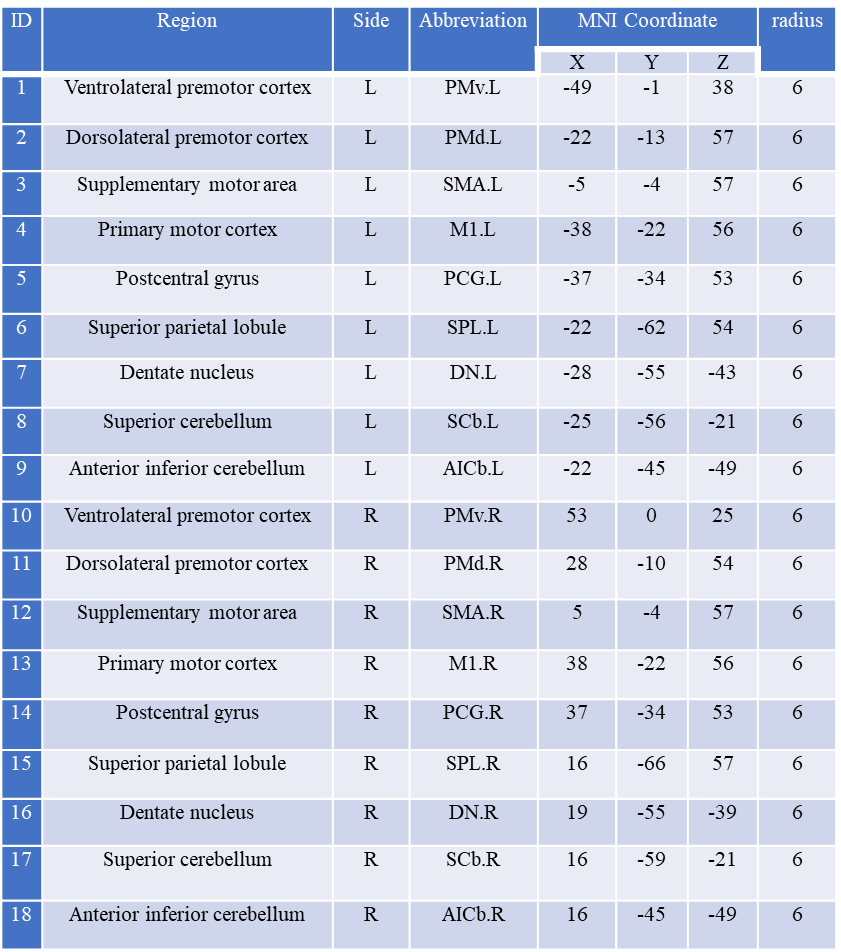

Supplement: Supplementary file 1 [file Table_1.DOCX]
